# Nicotine dependence in the mental disorders, relationship with clinical indicators, and the meaning for the user^1^

**DOI:** 10.1590/0104-1169.3549.2468

**Published:** 2014

**Authors:** Renata Marques de Oliveira, Antônio Carlos Siqueira, Jair Lício Ferreira Santos, Antonia Regina Ferreira Furegato

**Affiliations:** 2 Doctoral student, Escola de Enfermagem de Ribeirão Preto, Universidade de São Paulo, WHO Collaborating Centre for Nursing Research Development, Ribeirão Preto, SP, Brazil; 3 PhD, Professor, Faculdade de Medicina de Marília, Marília, SP, Brazil; 4 PhD, Full Professor, Faculdade de Medicina de Ribeirão Preto, Universidade de São Paulo, Ribeirão Preto, SP, Brazil; 5 PhD, Full Professor, Escola de Enfermagem de Ribeirão Preto, Universidade de São Paulo, WHO Collaborating Centre for Nursing Research Development, Ribeirão Preto, SP, Brazil

**Keywords:** Smoking, Schizophrenia, Mental Health, Psychiatric Nursing

## Abstract

**OBJECTIVE:**

to identify the degree of nicotine dependence among patients with schizophrenia and other mental disorders hospitalized in a general hospital, correlating these indices with clinical indicators and the meaning for the user.

**METHOD:**

the study was performed in the psychiatric unit of a general hospital, interviewing 270 patients with mental disorders using a questionnaire and the application of the Fagerstrom test. A descriptive statistical analysis of the data and thematic analysis of the content were performed.

**RESULTS:**

among the 270 patients with mental disorders, 35.6% were smokers; of whom, 53.2% presented high or very high nicotine dependence. Of the 96 smokers, 32 (33.3%) were schizophrenic, among whom, 59.4% presented high or very high dependence. Higher levels of dependence were also found among the 59 elderly people (61.5%) and 60 subjects with somatic comorbidities (62.5%). Meanings of smoking for the subjects: helps to forget problems and face daily conflicts; alleviates side effects of the medications; self-control; distraction; part of life.

**CONCLUSION:**

more intense tobacco dependence among schizophrenic patients is justified due to it helping them to cope with the difficulties of the disease. Nurses occupy a strategic position in the care.

## Introduction

Schizophrenia is a serious mental disorder that affects three out of every 10, 000 adults, of the global population. Despite the low incidence, the chronic course raises the prevalence to high levels, affecting 24 million people worldwide^(^ 1 ^)^. 

Although delusions and hallucinations are the more recognized symptoms of schizophrenia in the clinical practice, negative symptoms and cognitive deficits are mostly responsible for the impairment of the social functioning of these individuals, with damage to work skills and social interaction^(^ 2 ^-^ 3 ^)^. 

Epidemiological studies and meta analyzes agree that, in addition to psychosocial impairments, there are impairments of the physical health of individuals with schizophrenia (higher occurrence of cardiovascular and metabolic diseases and cancer) and decreased life expectancy. Tobacco use (eight in every 10) is one of the factors contributing to increased occurrences of crises, suicide attempts and need for hospitalization^(^ 4 ^-^ 7 ^)^. 

The high prevalence of smoking among individuals with schizophrenia draws attention to a historical moment in which tobacco use is being discouraged by the government and by various social segments. 

Although psychiatric care has undergone intense transformations in recent decades, with the development of pharmacological therapy, constructing a healthcare network capable of receiving the deinstitutionalized individual, little progress has been made in relation to the smoking habits of patients with mental disorders. There are few national studies on tobacco use by patients with mental disorders and the factors associated with smoking.

The recognition of the seriousness of tobacco use for individuals with schizophrenia causes nursing to question what can be done to change this context, since in addition to deinstitutionalization, the patient with mental disorders needs to be given the conditions to have a better quality of life^(^ 5 ^)^.

Nurses need to have technical and scientific knowledge about the effects of tobacco use among people with mental disorders, as this knowledge can be converted into care practices^(^ 8 ^)^.

Therefore, this study aimed to answer the following questions: 1) What is the meaning of smoking for patients with mental disorders hospitalized in the psychiatric unit of a general hospital? 2) Which variables are associated with smoking in this population? 3) Is there a difference in the degree of nicotine dependence, clinical indicators and meanings of smoking among schizophrenic patients and the patients with other disorders?

Aims: to identify the degree of nicotine dependence among patients with schizophrenia and other mental disorders, hospitalized in the psychiatric unit of a general hospital, correlating these indices with the clinical indicators (diagnosis, duration of disease, therapies, psychiatric hospitalizations, and somatic comorbidities) and the meaning for the user. 

## Method

This study used mixed convergent methods, answering the confirmatory and exploratory questions to the extent that the researchers could confirm their hypotheses through the hypothetical-deductive method (quantitative methodology) and generate new theories (induction) from the in-depth exploration of the phenomenon (qualitative methodology) and of the process by which the relationships between variables occur^(^ 9 ^)^.

This study was conducted in the psychiatric ward of a university, state hospital complex, in the state of São Paulo. The institution is the reference for secondary and tertiary levels for an estimated population of 1, 200, 000 inhabitants, living in 62 municipalities of the IX Regional Health Department (DRS IX). The psychiatric ward has 18 inpatient beds, with an mean occupancy of 15 patients per day and a mean hospital stay of 16 days.

A random probability sample was selected, which consisted of all the patients admitted to the psychiatric ward, from August 2010 to February 2012, with a mental and behavioral disorder diagnosis, and that agreed to participate in the study. Exclusion criteria: under 15 years of age; people diagnosed with mental retardation; users of alcohol and other drugs, without psychiatric comorbidities; people unable to communicate during the entire period of their hospitalization. 

**Figure 1:**
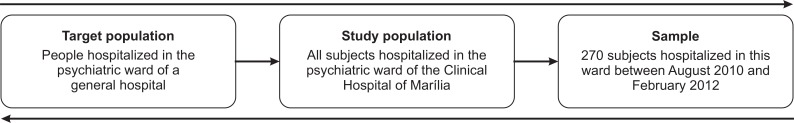
Description of sampling procedure and statistical inference

The minimum of patients with mental disorders that smoked estimated for the sample was 96 subjects (maximum error of 10% and 95% accuracy). Considering that preliminary surveys in this unit estimated that approximately one third of all the patients were smokers, the total sample size (smokers, abstinent smokers and nonsmokers) was 270 subjects (Figure 1). 

Individuals who used tobacco were considered smokers, regardless of the frequency of the use (daily and occasional smokers), the quantity used, and the length of time since beginning this practice, so as to cover both beginners and long-term smokers^(^ 10 ^)^.

This study was approved by the Research Ethics Committee (EERP/USP 1173/2010, CAAE 2009. 0.000.153-10). All the subjects signed the Terms of Free Prior Informed Consent.

Two instruments were used: 1) Smoker Identification Instrument for the Psychiatric Unit of a General Hospital (ITUP) and 2) Fagerstrom Test for Nicotine Dependence (FTND).

1) The ITUP is a questionnaire designed for this study, consisting of structured and semi-structured questions for smokers, abstinent smokers and nonsmokers. The structured questions aim to identify characteristics of the subjects: personal and sociodemographic (gender, age, education, marital status, living arrangement, origin, occupation, and income), clinical indicators (diagnosis, therapy received, psychiatric hospitalizations, and somatic comorbidities), and daily living habits (alcohol, coffee, illicit substances, games, television, physical exercise, reading, cultural activities, going out and traveling). Only the smokers responded to the semi-structured questions: duration of smoking; age at which smoking began; daily amount of cigarettes; monthly expenditure; attempts to quit smoking; whether the person feels able to quit smoking; effects of tobacco on the body and on the behavior; whether the person considers him/herself a smoker, and the meaning of tobacco in his/her life.

2) Fagerstrom Test for Nicotine Dependence (FTND), composed of six questions that classify the degree of dependency as very low, low, moderate, high, and very high. Validated in Brazil in 2002 (test-retest 0. 915 and Cronbach's α 0. 642)^(^ 11 ^)^. 

A total of 270 patients with mental disorders were interviewed and their responses were recorded using the form. The responses to the semi-structured questions were recorded and transcribed.

The statistical analysis of the quantitative data was performed using the Stata program (version 10. 10), with the application of tools of descriptive statistics and bivariate analysis (Fisher's exact test or chi-square test, with maximum probability of error - alpha of 5%).

The qualitative data (content expressed by the subjects) underwent thematic analysis to identify the effect of tobacco use for the patients with mental disorders. Frequencies were calculated for the different meanings identified. 

In convergent mixed methods, the quantitative and qualitative data were analyzed independently, however, with the triangulation of the information in the discussion^(^ 9 ^)^.

## Results

### Characterization of the personal, sociodemographic data and clinical indicators of the subjects 

Of the 270 patients with mental disorders of this study, 91 (70.7%) were women. The participants had a mean age of 38.9 years (15-88); 235 (87%) had completed elementary or high school education, and 35 (13%) higher education; 108 (40%) were single, 105 (38.9%) married, 37 (13.7%) separated or divorced, and 20 (7.4%) widowed; 126 (46.7%) were living without a partner, but with family; 184 resided in the city under study; 176 (65.2%) had some type of occupation; and the median family income was R$ 1,000.00.

Of the subjects studied, 226 (83.7%) had schizophrenia, mood and personality disorders; 62 (23%) subjects had not been receiving psychiatric monitoring, prior to the current hospitalization, 128 (47.4%) had received medication only, and 80 (29.6%) had received medication in association with other interventions (psychological counseling, support groups, occupational therapy). A total of 136 patients (50.7%) were hospitalized for the first time.

### Characterization of the activities and habits of daily life of the subjects 

Among the 270 patients with mental disorders of the study, 96 (35.6%) reported using tobacco. The length of time from the start of smoking until that moment, ranged from 3 days to 52 years. Of the 96 smokers, 50 (52.1%) began smoking between 11 and 15 years of age. At that time, they smoked a mean of 24.2 cigarettes per day (1-100 cigarettes) [standard deviation (s=19 cigarettes/day)] and spent a mean of R$ 86.00 per month on tobacco.

Table 1 presents the frequency of the activities and habits of daily life, reported by the 270 patients with mental disorders. It can be seen that activities and habits considered healthy, such as physical exercise, reading, cultural activities and traveling, had a low frequency. 

**Table 1:** Activities and habits of daily life of the 270 patients with mental disorders. Marilia, SP, Brazil, 2012

Activities and habits of daily life	n	%
Television	215	79.6
Coffee	200	74.1
Going out	143	53
Reading	130	48.2
Physical exercise	79	29.3
Traveling	75	27.8
Alcohol	44	16.3
Cultural activities	40	14.8
Games	21	7.8
Illicit substances	13	4.8

### Degree of nicotine dependence and its association with the variables studied 

The Fagerstrom Test for Nicotine Dependence (FTND) was applied with the 96 patients with mental disorders that reported using tobacco. Fifty-one subjects (53.2%) were classified as having a high or very high level of dependence, 13 (13.5%) a moderate level, 18 (18.8%) a low level, and 14 (14.6%) with a very low level of nicotine dependence.

Of the 96 smokers, 32 (33.3%) were schizophrenic, among whom 59.4% (χ^2^=39.0037, Pr=0.027) had a high or very high level of dependence.

Higher levels of nicotine dependence (high and very high) were also found among the elderly patients (61.9%, Fisher 0.006) and those with somatic comorbidities - cardiovascular, digestive, endocrine, respiratory (62%, Fisher 0.048).

Furthermore, it was found that the smokers more regularly used alcohol (Fisher 0.006), illicit substances (Fisher 0.001) and coffee (Fisher 0.000), with no evidence of an association between the level of nicotine dependence and these variables (Fisher's exact test: alcohol 0.837; illicit substances 0.523; coffee 0.380). Some variables associated with the level of nicotine dependence are presented in Table 2.

**Table 2:** Variables associated with the level of nicotine dependence of the 96 patients with mental disorders. Marilia, SP, Brazil, 2012

Variables associated with smoking		Level of nicotine dependence					
		Very Low n(%)	Low n(%)	Moderate n(%)	High n(%)	Very high n(%)	Total n(%)
Cigarettes/day							
	1-10	11(78.6)	7(38.9)	4(30.8)	1(3.6)	1(4.4)	24(25.0)*
	More than 11	3(21.4)	11(61.1)	9(69.2)	27(96.4)	22(95.7)	72(75.0)
Smoking duration							
	≤20 years	11(78.6)	9(50.0)	7(53.9)	14(50.0)	5(21.7)	46(47.9)^†^
	≥21 years	3(21.4)	9(50.0)	6(46.2)	14(50.0)	18(78.3)	50(50.1)
Monthly spending							
	None	3(21.4)					3(3.1)^‡^
	≤R$60	9(64.3)	15(83.3)	10(76.9)	16(57.1)	2(8.7)	52(54.2)
	>R$60	2(14.3)	3(16.7)	3(23.1)	12(42.9)	21(91.3)	41(42.7)
Able to quit							
	Yes	14(100)	12(66.7)	7(53.9)	12(42.7)	9(39.1)	54(56.3)^§^
Tried to quit							
	Yes	8(57.1)	17(94.4)	12(92.3)	21(75.0)	23(100)	81(84.4)^§^
Effects on the body							
	Yes	12(85.7)	18(100)	13(100)	28(100)	23(100)	94(97.9)^||^
Effects on the behavior							
	Yes	12(85.7)	18(100)	12(92.3)	28(100)	23(100)	93(96.9)^¶^
Considers oneself a smoker		5(35.7)	14(77.8)	9(69.2)	25(89.3)	21(91.3)	74(77.1)**
Total		14(100)	18(100)	13(100)	28(100)	23(100)	96(100)

* Fisher test - significant result

* 0.000

† Fisher test - significant result

† 0.016

‡ Fisher test - significant result: 0.000

§ Fisher test - significant result: 0.002

|| Fisher test - significant result: 0.037

¶ Fisher test - significant result: 0.026

** Fisher test - significant result: 0.001

### The meaning of tobacco use for the smokers (S) with mental disorders 

The majority of the smokers believed that tobacco use among psychiatric patients is different from that of other people: 75 (78.1%) believed that psychiatric patients smoke more cigarettes than the general population and 69 (71.9%) believed that the psychiatric population find it more difficult to quit. 

There was higher proportion of subjects among the schizophrenic patients, 30 (93.8%), who believed that psychiatric patients smoke more cigarettes than other people (Fisher 0.036), a higher proportion in relation to each of the other disorders, as well as compared to the total sample of smokers. The greater difficulty to quit smoking was also positively associated with a higher frequency of previous psychiatric hospitalizations (Fisher 0.042).

In the statements of the 96 smokers with mental disorders, three categories were identified, related to the meanings attributed to tobacco use by the psychiatric patients: The cigarette is a support to face the difficulties originating from the mental disorder; Smoking alleviates the psychiatric symptoms and side effects of the medication; Smoking gives pleasure. 

### Category 1 - The cigarette is a support to face difficulties originating from the mental disorder

Tobacco, according to 31.3% of the psychiatric patients, serves as support to forget the everyday problems and, according to 3.1%, to cope with the stigma.

*Smoking is an escape valve. We need to take refuge in something. *(S95)

*The most difficult thing is the discrimination of the people. I'm afraid to go outside, but I face it with the cigarette. *(S66)

Some subjects (5.2%) considered tobacco irreplaceable and necessary for life.

*I have still not found anything that can replace the cigarette. *(S28)

*Somehow, it *[the cigarette]* supplies something here. *(S72)

For those patients without motivation to continue living, tobacco is seen as a form of self-destruction (6.3%).

*I have no more will to live, so for me it does not matter. *(S50)

### Category 2 - Smoking alleviates the psychiatric symptoms and side effects of the medication 

The majority of the smokers (78.1%) used tobacco to self-medicate the psychiatric symptoms. Some (5.2%) reported that cigarette smoking increases self-control and decreases impulsivity.

*The cigarette, for me, the way I'm depressed, it became a kind of medicine. *(S61)

*Cigarettes help decrease impulsivity. *(S29)

A total of 79% of the smokers believed that smoking reduces anxiety, 57.3% that it improves the mood, and 29.2% and that improves concentration.

Tobacco was also used to alleviate the side effects of the psychiatric medications (4.2%).

*The drugs make me a little sleepy, so I'll go and smoke. *(S14)

### Category 3 - Smoking gives pleasure

Tobacco was used for pleasure (28.1%) and as a distraction to pass the time (18.8%). 

*Just the pleasure of smoking is enough not to quit. The cigarette gives me pleasure that I don't find in other things.* (S28)

*When I have nothing to do I smoke. It's a way to pass the time. *(S66)

Some participants recognized the cigarette as a companion in times of loneliness (14.6%) and as a facilitator of social interactions (6.3%).

*The cigarette is a friend, a companion. Even when I'm alone it is my companion. It cannot talk, but it is always there with me. *(S70)

*When we smoke it seems like the conversation flows. It seems like you have the strength to continue talking, to be freer, more secure.* (S46)

Frequency of the meanings attributed to tobacco use by the schizophrenic patients: helps you to forget problems (46.7%); alleviates the side effects of the psychiatric medications (50%); self-control (60%); distraction to pass the time (44.4%); coping with stigma (66.7%); irreplaceable/necessary for life (80%). 

In the statements of the patients with mood disorders, the more frequent meanings of tobacco use were: self-destruction (50%); a companion in times of loneliness (42.9%); a facilitator of social interactions (66.7%); pleasure (40.7%); and irreplaceable/necessary for life (50%). The schizophrenic patients and those with personality disorders also presented high frequencies in these aspects. 

The patients with mental disorders and a high or very high level of nicotine dependence were those that most reported tobacco use as an aid to forget problems (53.4%), self-destruction (66.6%), a companion in times of loneliness (57.1%), a facilitator of social interactions (66.6%) and as irreplaceable and necessary for life (80%).

## Discussion

This study supports the hypothesis that tobacco use is more common among individuals with mental disorders (by approximately one third) than in the other groups of the population, given that the current prevalence of smoking in the Brazilian population is 17.5%^(^ 12 ^)^. 

Studies conducted in Australia, United States and Israel show that the frequency of smoking among patients with mental disorders is approximately twice that found in people with no history of psychiatric illness^(^ 13 ^-^ 15 ^)^.

Although one third of the subjects in this study were smokers, this information was not registered in any patient records, despite nicotine dependence being a diagnosis recognized by the ICD 10 or DSM IV. Studies in the United States and Switzerland, also on the absence of smoking registration in patient records, suggest that professionals do not worry about tobacco use in psychiatric hospitalization^(^ 13 ^,^ 16 ^)^. 

In this study, an association was found between the use of tobacco, illicit substances and alcohol. A study with 745 schizophrenic patients hospitalized in psychiatric services of the state of Maryland, United States, found that those who were using large amounts of cigarettes per day were more likely to use alcohol and illicit substances. The association between tobacco and these substances is worrying because it increases the risk of the psychiatric patient developing somatic complications (cardiac, respiratory, hepatic, gastrointestinal and neurological), as well as changes in the behavior and sleep pattern^(^ 17 ^)^.

The results of the present study showed that tobacco dependence is more intense among individuals with schizophrenia, confirming the results of a meta-analysis performed with studies from 20 countries. The meta-analysis also revealed that lower rates of the discontinuation of tobacco use are found among schizophrenic patients^(^ 4 ^)^. Recent studies conducted in the United States, Israel and Brazil also showed high levels of nicotine dependence among individuals with schizophrenia^(^ 17 ^-^ 19 ^)^.

The high level of nicotine dependence among subjects with somatic comorbidities is consistent with the knowledge that smoking is a risk factor for physical complications. A Brazilian study, with 100 patients who suffered acute myocardial infarction, showed that the smokers had a greater perception of the risk of becoming ill, as they sought professional help faster than the other subjects when faced with the myocardial infarction symptoms^(^ 20 ^)^.

The subjects with higher nicotine dependence, in the present study, felt less able to quit smoking. A study of 200 psychiatric patients in the United States revealed that the individuals with schizophrenia and schizoaffective disorder felt less motivated to quit smoking than the other subjects^(^ 21 ^)^. 

An Australian study revealed that the patients with psychotic disorders used tobacco to confront the challenges originating from the mental disorder and the pharmacological therapy - relief from the collateral effects of the medications, reduction of the symptoms, and for pleasure and relaxation^(^ 22 ^)^. Although the psychiatric patients with more severe tobacco dependence are the ones who perceive the effects of smoking on their bodies and behavior, they are also the ones with a higher frequency of failed attempts to quit. 

In some of the patient records of the subjects of this study, there were records reminding the nursing team to warn smokers about the times that smoking is allowed. This is recognition of the participation of nursing in the culture of smoking in psychiatric hospitalizations. 

The continuous care provided by the nursing team, in the inpatient psychiatric units, allows them to become closer to the patients. Nursing actions, aimed at reducing smoking and promoting a healthier lifestyle among patients with mental disorders should be a priority.

An Australian study recommends that psychiatric patients should be given assistance to find resources that help them cope with the mental disorder. Interventions for the abandonment of substance use, including tobacco, may have more success when included in the therapeutic plan^(^ 22 ^-^ 23 ^)^. 

Nurses are key players in the multidisciplinary team, as they accompany the patients and know their behavior well, which can be used to help the patients become aware of the resources they have to cope with the disorder and the tobacco use. For this, nurses need to be prepared and willing to collaborate.

## Conclusions

A higher level of nicotine dependence was associated with older age, the diagnosis of schizophrenia, and the presence of somatic comorbidities. Both the schizophrenic patients and the subjects with higher levels of dependence used tobacco to cope with the difficulties arising from the mental disorder - they reported that tobacco helps them to forget their problems and to confront everyday conflicts, provides relief from the side effects of the medication, and helps with self-control. They also consider tobacco to be part of their lives. 

Tobacco use by schizophrenic patients is a challenge for psychiatry. Nurses are in a strategic position to intervene in this context, with preventive and rehabilitative actions in the therapeutic plan of each patient under their care.
